# CD8+ T Cells Protect During Vein Graft Disease Development

**DOI:** 10.3389/fcvm.2019.00077

**Published:** 2019-06-13

**Authors:** Karin H. Simons, Margreet R. de Vries, Hendrika A. B. Peters, J. Wouter Jukema, Paul H. A. Quax, Ramon Arens

**Affiliations:** ^1^Einthoven Laboratory for Experimental Vascular Medicine, Leiden University Medical Center, Leiden, Netherlands; ^2^Department of Surgery, Leiden University Medical Center, Leiden, Netherlands; ^3^Department of Cardiology, Leiden University Medical Center, Leiden, Netherlands; ^4^Department of Immunohematology and Blood Transfusion, Leiden University Medical Center, Leiden, Netherlands

**Keywords:** T cell, co-stimulation, CD8+ activation, vein graft disease, vein graft failure, TCR–T cell receptor, costimulatary pathway

## Abstract

**Aims:** Vein grafts are frequently used conduits for arterial reconstruction in patients with cardiovascular disease. Unfortunately, vein graft disease (VGD) causes diminished patency rates. Innate immune system components are known to contribute to VGD. However, the role of T cells has yet to be established. The purpose of this study was to investigate the role of T cells and T cell activation pathways via the T cell receptor (TCR), co-stimulation and bystander effect in VGD.

**Methods and results:** Here, we show upon vein graft surgery in mice depleted of CD4+ T cells or CD8+ T cells, that CD8+ T cells are locally activated and have a major protective role for vein graft patency. In presence of CD8+ T cells vein grafts appear patent while CD8+ T cell depletion results in occluded vein grafts with increases apoptosis. Importantly, the protective effect of CD8+ T cells in VGD development was TCR and co-stimulation independent. This was demonstrated in vein grafts of OT-I mice, CD70^−/−^, CD80/86^−/−^, and CD70/80/86^−/−^ mice compared to C57BL/6 mice. Interestingly, cytokines including IL-15, IL-18, IL-33, and TNF are up-regulated in vein grafts. These cytokines are co-operatively capable to activate CD8+ T cells in a bystander-mediated fashion, in contrast to CD4+ T cells.

**Conclusions:** T cells are modulators of VGD with a specific protective role of CD8+ T cells, which are locally activated in vein grafts. CD8+ T cells may protect against occlusive lesions by providing survival signals, and concert their protection independent of TCR and co-stimulation signaling.

## Introduction

The vena saphena magna is the preferred conduit for arterial reconstruction in patients with cardiovascular disease. After vein graft surgery, intimal hyperplasia development through proliferation, and migration of vascular smooth muscle cells (VSMCs), deposition of extracellular matrix, and macrophage accumulation and proliferation, leads to narrowing of the vessel lumen, called vein graft disease (VGD) (1). Therefore, vein grafts display limited patency, with a rate of occlusion of ~60% in 10 years after surgery. Whereas, the innate immune system has been shown to be involved in VGD (2), the role of T cells of the adaptive immune system has yet to be established.

Previous studies have shown that T cells play a role in other cardiovascular diseases such as atherosclerosis (3, 4). Both CD4+ and CD8+ T cells are present in atherosclerotic plaques. Th1 CD4+ T cells have pro-atherogenic effects via production of IFNγ and TNFα, while Th2 CD4+ T cells and regulatory T cells limit atherosclerotic plaque formation via IL-10 and TGFβ production (5–8). Remarkably, CD8+ T cells seem to be locally activated in atherosclerotic plaques (9). CD8+ T cells have demonstrated both protective and pathogenic roles in atherosclerosis (10, 11). Whether CD4+ and CD8+ T cells are positive or negative modulators of VGD, is yet unknown.

T cells can be activated via different stimulatory signals provided by antigen presenting cells (APCs). Antigenic stimulation (signal 1) occurs via the T cell receptor (TCR) upon binding cognate peptide-MHC complexes. The Ig family member CD28 is constitutively expressed on naive T cells (TN), and provides imperative co-stimulation (signal 2) that cooperates with TCR triggering to achieve proper T cell activation. The ligands of CD28, i.e., CD80 and CD86, are up-regulated upon activation of APCs. Interaction of CD80/86 with the inhibitory receptor CTLA-4 leads to dampening of T cell activation (12–14). The co-stimulatory receptor CD27, belonging to the TNFR family, is also present on the majority of T cells (15), and interacts with its unique ligand CD70. Expression of CD70 is transiently up-regulated on subsets of activated APCs, and this constricted regulation is essential to avoid immune over-activation (16, 17). Inflammatory cytokines such as IL-12, IL-18, and type I interferons (IFN) can provide additional bystander signals (signal 3) for T cell activation (18). Macrophages and dendritic cells are well-studied APCs known to provide all these important signals to T cells, and both are present in vein grafts (1, 19). Notably VSMCs are also able to express MHC molecules and co-stimulatory molecules, especially after stimulation with interferon-γ (IFNγ) (20, 21), and in this way, VSMCs might be capable of T cell activation and function as APCs in vein grafts.

In this study, we examined the role of T cells in the development of VGD, and dissected the underlying mechanism of T cell activation pathways involved. Investigations in a murine VGD model revealed a strong protective effect of locally activated CD8+ T cells by depletion of either CD4+ T cells or CD8+ T cells. This protective effect was associated with increased apoptosis in CD8+ T cell depleted mice. Remarkably, by using TCR transgenic mice and co-stimulation knockout mice, we showed that this protective effect of CD8+ T cells in VGD development is TCR and co-stimulation independent. In addition, multiple bystander cytokines are present in the vein grafts and are able to induce CD8+ T cell activation. Thus, CD8+ T cells protect VGD development in a TCR independent and co-stimulation independent way, but most likely via bystander cytokines.

## Materials and Methods

### Study Approval and Mice

This study was performed in compliance with Dutch government guidelines and the Directive 2010/63/EU of the European Parliament. All animal experiments were approved by the animal welfare committee of the Leiden University Medical Center. C57BL/6 mice were purchased from Charles River Laboratories and crossbred in our own colony for at least 18 generations. CD70^−/−^, CD80/86^−/−^, and CD80/86/70^−/−^ were bred in house on a C57BL/6 background (22). T cell receptor (TCR) transgenic mice recognizing the MHC class I-restricted SIINFEKL epitope derived from Ovalbumin (OT-I mice) (23) were obtained from The Jackson Laboratory. The TCR transgenic OT-I mouse is known to have an almost exclusive monoclonal TCR population (Va2/Vb5) (24). This is caused by the strong transgenic TCR expression, which accelerates passing of T cells via the double positive (DP) stage resulting in negligible endogenous segments to be rearranged. The transgenic beta chain induces allelic exclusion and suppresses rearrangement of endogenous beta chains. In TCR beta chain transgenic mice ~90% of peripheral T cells carry the transgenic beta chain and only 0–20% expresses endogenous ones. In the OT-I TCR transgenic mice we used, 99.6% of the CD8+ T cells had a Va2(Vb5) TCR (Figure S1) and 0.021% of CD8+ T cells expressed endogenous TCRs (Vb13 TCR), which we showed in the spleen. Thus, the endogenous TCR is barely present in OT-I TCR transgenic mice. All used mice were male mice aged 10 to 18 weeks old.

### Vein Graft Surgery

Vein graft surgery was performed in mice by donor caval vein interpositioning in the carotid artery of recipient mice (25). All recipient mice received donor caval veins from genetic identical littermates. After 28 days mice were sacrificed and blood, draining, and non-draining lymph nodes, spleen, and vein graft or vena cava were harvested for flow cytometric analysis and/or (immuno)histochemical analysis. Mice were anesthetized by intraperitoneal injection of a combination of midazolam (5 mg/kg, Roche), medetomidine (0.5 mg/kg, Orion), and fentanyl (0.05 mg/kg, Janssen) before surgery and at sacrifice. Flumazenil (0.7 mg/kg, Fresenius Kabi) was intraperitoneal injected after surgery to antagonize anesthesia. After surgery and on indication, buprenorphine (0.1 mg/kg, MSD Animal Health) was given as an analgesic. Patency of vein graft was macroscopically determined at sacrifice and stated as clearly pulsatile, not clearly pulsatile or occluded.

### Gene Set Enrichment Analysis

Vein grafts of male mice were harvested 14 days after surgery and compared with vein grafts harvested at surgery (day 0) (*n* = 4/group). Total RNA was isolated from vein grafts using TRIzol^®^ (Ambion^®^ by Life Technologies) and was quantitated using a NanoDrop 1,000 Spectrophotometer (Thermo Scientific). cDNA was synthesized using a High-Capacity cDNA Reverse Transcription Kit (Applied Biosystems) according to the manufacturer's protocol.

Gene set enrichment analysis (GSEA) was performed with the curated gene sets from Kegg, Biocarta, the Reactome, and published studies, resulting in a total of 1,564 gene sets. For each gene set an enrichment score (ES) is calculated representing the difference between expected and observed ranking, which correlates with the phenotype of the vein grafts. By permuting the phenotype labels, a statistical significance (nominal *P-*value) for the ES is provided. An adjustment for the gene set size generates the normalized enrichment score (NES). To correct for multiple testing the proportion of false positives is calculated to provide the false discovery rate (FDR) corresponding to each NES. A FDR <5% was considered significant. Method is fully described previously (26).

### *In vivo* CD4+ and CD8+ T Cell Depletion

Depletion of either CD4+ T cells, CD8+ T cells or both was performed by intraperitoneal injections of depleting antibodies. Male mice were divided in five groups; CD4+ T cell depletion group (*n* = 12), CD8+ T cell depletion group (*n* = 12), CD4+, and CD8+ T cell depletion group (*n* = 12), control group (*n* = 12), and a naive (not operated group) group (*n* = 7). At 1 day prior to operation and 3, 10, 17, and 24 days after operation mice were injected with antibodies to deplete CD4 T cells (anti-CD4 clone GK1.5), CD8+ T cells (anti-CD8 clone 2.43), or both CD4+ T cells and CD8+ T cells or were injected with a control antibody (control mAb clone GL113). Prior to operation the injected amount was 200 μg mAb per mouse and, post-operative 100 μg per mouse was provided. Blood from tail vein was obtained 7 and 14 days after surgery and at sacrifice to check T cell subset depletion with flow cytometry.

### Vein Graft Mouse Experiments

To examine if donor caval veins, used as a vein graft, contain (activated) T cells prior to operation, we performed vein graft surgery in male C57BL/6 mice (*n* = 3) and harvested the vein graft after 28 days, or performed no surgery (*n* = 3), and harvested the caval vein after 28 days. Vein grafts or caval veins were used for flow cytometry.

Vein graft surgery was performed in CD80/86/70^−/−^ mice (*n* = 14), CD80/86^−/−^ mice (*n* = 14), CD70^−/−^ mice (*n* = 12), and C57BL/6 mice as a control (*n* = 11), fed a chow diet *ad libitum* and sacrificed after 28 days. Vein grafts were harvested for immunohistochemical analysis.

Vein graft surgery was performed in male OT-I mice (*n* = 5) and male C57BL/6 mice as a control (*n* = 11). Vein grafts were used for flow cytometry.

### Flow Cytometry

Flow cytometry was performed on blood, spleen, draining, and non-draining lymph nodes, vena cava, and/or vein grafts. Single-cell suspensions were prepared from spleens and draining and non-draining lymph nodes, by mincing the tissue through a 70 μm cell strainer (BD Biosciences). Vein grafts and vena cavae were pre-treated with a liberase enzyme mix for 30 min and washed with 10 ml Iscove's Modified Dulbecco's Medium (IMDM, Lonza), 8% fetal calf serum (FCS, Life Technologies) and 100 U/mL penicillin/streptomycin (PS, Life Technologies). Erythrocytes were lysed in a red blood cell lysis buffer (hypotonic ammonium chloride buffer). Approximately 400,000 harvested cells from blood, spleen, draining, and non-draining lymph nodes and ~40,000 harvested cells from vena cava and vein grafts were used for the flow cytometry. Conjugated monoclonal antibodies to mouse CD11b (V450, eBioscience), Class II (V500, BD Horizon), Ly6C (fluorescein isothiocyanate [FITC], Biolegend), CD11c (phycoerythrin [PE], Biolegend), CD86 (Allophycocyanin [APC], Biolegend), F4/80 (PE-Cy7, Biolegend), Ly6G (Alexa Fluor 700, Biolegend), CD19 (APC-Cy7, eBioscience), CD44 (V450, eBioscience), CD8 (FITC, Biolegend), TCR Beta (PE, eBioscience), CD25 (APC, eBioscience), KLRG1 (PE-Cy7, eBioscience), CD62L (APC-Cy7, eBioscience), CD4 (Brilliant Violet 605, Biolegend) were used. Dead cells were excluded by positivity for 7-aminoactinomycinD (7-AAD) (Invitrogen). Flow cytometric acquisition was performed on a BD LSR II flow cytometer (BD Biosciences). Data were analyzed using FlowJo V10.1 software (BD). Flow cytometry gating strategies are shown in Figure S2.

### RNA Isolation, cDNA Synthesis, and RT-PCR From Vein Graft Tissue

Total RNA was isolated from 10 (20 μm thick) paraffin sections of vein grafts 3, 7, 14, and 28 days after surgery. The method and analysis of RNA isolation, cDNA synthesis, and RT-PCR was performed as previously described (27). In brief, RNA was isolated according to manufacturer's protocol (FFPE RNA isolation kit, Qiagen). FFPE RNA was reverse transcribed using the RT^2^ First Strand Kit (SA Biosciences). RNA for single qPCR was reverse transcribed using a High-Capacity cDNA Reverse Transcription Kit according to the manufacturer's protocol. Commercially available TaqMan gene expression assays for hypoxanthine phosphoribosyl transferase (HPRT1), and selected genes of interest were used (Applied Biosystems). qPCR analysis was performed using a RT^2^ Profiler PCR Array (SA Biosciences) according to the manufacturer's protocol. The complete list of the genes analyzed is available at https://www.qiagen.com/us/products/discovery-and-translational-research/pcr-qpcr/qpcr-assays-and-instruments/mrna-incrna-qpcr-assays-panels/rt2-profiler-pcr-arrays/?catno=PAMM-016Z#geneglobe. Prior to RT^2^ profiler array, all qPCRs were performed on a 7500/7500 Fast Real-Time PCR System. In total we used six vein grafts per group, two pools of three vein grafts were analyzed. With the average of these two pools, the 2-ΔΔCt method was used to analyse the relative changes in gene expression. The fold change compared to the control vein graft was measured.

### Vascular Wall Lesion Analysis

Vein graft samples were embedded in paraffin and sequential cross sections (5 μm thick) were made throughout the embedded vein grafts. To quantify the vein graft thickening (intimal hyperplasia), HPS staining was performed using Hematoxylin, Phloxin 0.25%, and Saffron 0.3%. Vessel wall area (vein graft thickening) was defined as the area between lumen and adventitia and determined by subtracting the luminal area from the total vessel wall area. Antibodies directed at Mac-3 (BD Pharmingen) to quantify macrophages and alpha smooth muscle cell actin (αSMactin, Sigma) to quantify smooth muscle cells were used for immunohistochemical staining (28). Sirius red staining (Klinipath 80115) was performed to quantify the amount of collagen present in the vein grafts. The immuno-positive area measured is expressed as a percentage of the vessel wall area. Stained slides were photographed using microscope photography software (Axiovision; Zeiss) and image analysis software was used (Qwin; Leica, Wetzlar). Fluorescent double staining was performed to identify the presence of CD3+ T cells. Vein grafts harvested after 28 days of C57BL/6 mice were stained with CD3+ (Abcam). Slides were covered with Invitrogen Diamond Antifade Mountant (Invitrogen) with 4',6-diamidino-2-phenylindole (DAPI). Fluorescent double staining were acquired with the fluorescent slide scanner (3DHistech).

### Cell Culture

Vascular smooth muscle cells were acquired by isolating them from male mouse aortas (29). VSMCs were cultured using medium consisting of DMEM Glutamax with 20% fetal bovine serum (FBS, Sigma), 1% non-essential amino acids, and 1% PS.

D1 cells are a long-term growth factor-dependent immature myeloid dendritic cell line of splenic origin derived from a female C57BL/6 mouse. D1 cells were cultured in IMDM containing 10% heat inactivated FBS, 2 mM GlutaMax (GIBCO), 50 μM β-mercaptoethanol and 30% supernatant from R1 cells (mouse fibroblast NIH/3T3 cells transfected with mouse GM-CSF gene), which was collected from confluent cultures and filtered.

### *In vitro* CD8+ T Cell Activation

CD8+ T cells of OT-I mice were isolated from the spleen of male and female mouse, by using the mouse CD8+ T lymphocyte enrichment set (BD Biosciences). VSMC ability to activate T cells was measured in supernatant of 50,000 VMSCs cultured with 500,000 CD8+ T cells of OT-I mice, and stimulated for 24 h with Ovalbumin (Ova) and lipopolysaccharide (LPS) 100 ng/mL from *Escherichia coli* K-235 (Sigma-Aldrich). DCs (as described above) were used as positive control. As VSMC control groups, VSMCs were cultured without OTI T cells or without Ovalbumin, or OTI T cells were stimulated with Ovalbumin without VSMCs.

To test the impact of cytokine combinations, 100,000 CD8+ T cells or CD4+ T cells isolated from spleens of C57BL/6 mice, were stimulated for 24 h with agonistic antibodies against CD3 (0.1 μg/ml), or with the following cytokines (either single or combined): IL-2 (20 ng/ml), IL-18 (20 ng/ml), IL-12 (20 ng/ml), IL-33 (20 ng/ml), IL-15 (100 ng/ml), and TNFα (20 ng/ml). Culture medium was used as a control. In all experiments, the concentration of IFNγ was measured in supernatant using ELISA assays (BD Biosciences).

### Statistical Analysis

All data are presented as mean ± SEM. GraphPad Prism 7.0 software was used for statistical analyses. Analysis of parametric data, that passed normality and equal variance tests, were performed by using a 2-tailed Student's *t*-test to compare individual groups and a 2-way ANOVA comparing more than two groups. Mann-Whitney test was used for non-parametric data to compare individual groups that did not pass normality and equal variance tests, and a Kruskal-Wallis test was used to compare more than two groups. *P* < 0.05 was considered significant.

## Results

### Presence of Inflammatory Cells in Vein Grafts

**Figure 1:**
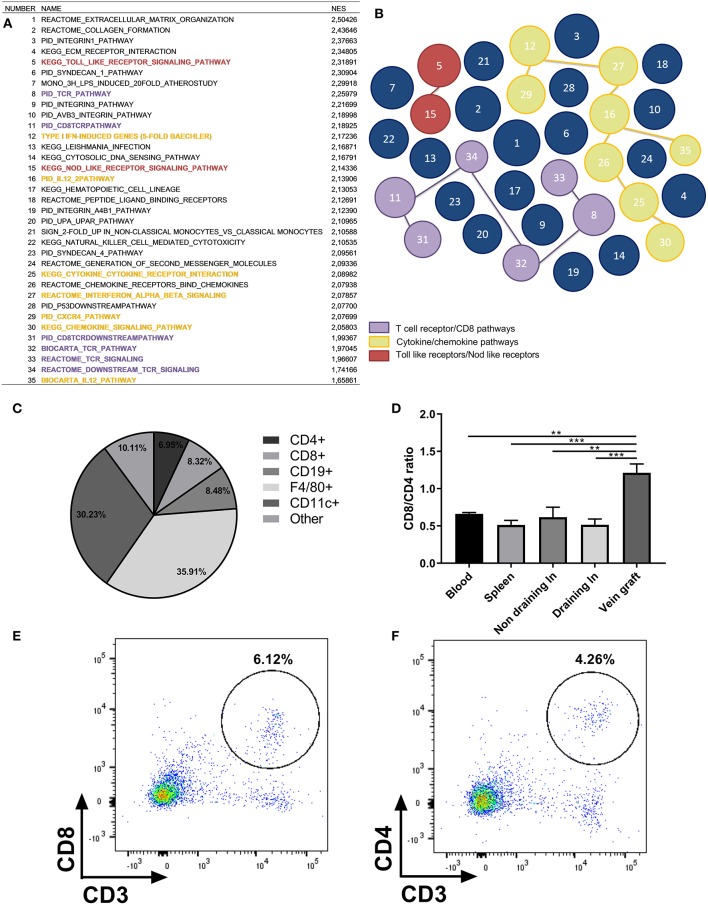
Presence of inflammatory cells in vein grafts. **(A)** Pathway analysis by gene set enrichment analysis (GSEA) on 1,564 available gene sets. The top 35 of significantly up-regulated gene sets found in vein grafts sacrificed after 14 days compared to vena cavae at day 0 are shown. Normalized enrichment score (NES) is shown. For each gene set (pathway) an enrichment score (ES) is calculated that represents the difference between expected and observed rankings which correlate with the phenotype. An adjustment for the gene set size generates the normalized enrichment score (NES). Colors are similar to the illustration in figure. **(B)** With TCR/CD8 pathways in purple, cytokine/chemokine pathways in yellow and Toll like receptors/Nod like receptors in red. The bigger the size of the circle, the higher the up-regulation of the gene. **(C)** Vein graft cell composition measured with FACS analysis. Percentage of CD4+, CD8+, CD19+, CD11b+, and remaining cells in vein grafts of C57BL/6 male mice as a percentage of all hematopoietic cells is shown, *n* = 4. **(D)** Ratio of CD8+/CD4+ T cells in vein grafts, blood, spleen, and draining lymph node is shown, *n* = 4. CD8+ and CD4+ T cells are a percentage of total CD3+ T cells. Significant differences between blood, spleen, (non) draining lymph nodes compared to vein grafts are indicated. **(E)** Example of flow cytometry plot is shown of CD3+ CD8+ T cells in vein grafts, and **(F)** CD3+ CD4+ T cells in vein grafts. ^**^P < 0.01, ^***^ *P* < 0.001, one-way ANOVA.

To examine whether genes of the adaptive immune system are actively transcribed in vein grafts, we performed pathway analysis by GSEA on 1,564 available gene sets. We identified 176 significantly up-regulated curated gene sets in vein grafts sacrificed after 14 days compared to vena cavae at day 0, whereas 52 gene sets were down-regulated. In the top 35 of significantly up-regulated gene sets found in vein grafts, TCR and CD8+ T cell pathways are found besides the expected extracellular matrix organization pathways (Figures 1A,B). Cytokine/chemokine pathways and Toll and Nod-like receptors are also found among the most up-regulated gene sets, illustrating that pathways of the immune system are active in the development of VGD.

To show that immune cells are present in vein grafts, we stained vein grafts with the lymphocyte marker CD3. We showed that infiltrating CD3+ T cells are present in vein grafts (Figure S3). In addition, we analyzed vein grafts compared to blood, non-draining lymph nodes, draining lymph nodes, and spleens with flow cytometry. Both CD4+ and CD8+ T cells are abundantly present in vein grafts. Interestingly, CD8+ T cells constitute 8.32% ± 3.15 while the percentage of CD4+ T cells is 6.95% ± 2.13 (Figure 1C). An elevated CD8/CD4+ T cell ratio in vein grafts was not observed in other organs (Figures 1D–F). Other abundant immune subsets in vein grafts are F4/80+ macrophages (35.91% ± 2.83), CD11c+ dendritic cells (30.22% ± 1.44), and CD19+ B cells (8.48%±1.58). Thus, compared to lymphoid organs such as spleen and draining and non-draining lymph nodes (Figure S4), vein grafts have a higher CD8/CD4+ T cell ratio, relatively fewer B cells and more myeloid cells.

### T Cells Are Locally Activated in Vein Grafts

**Figure 2:**
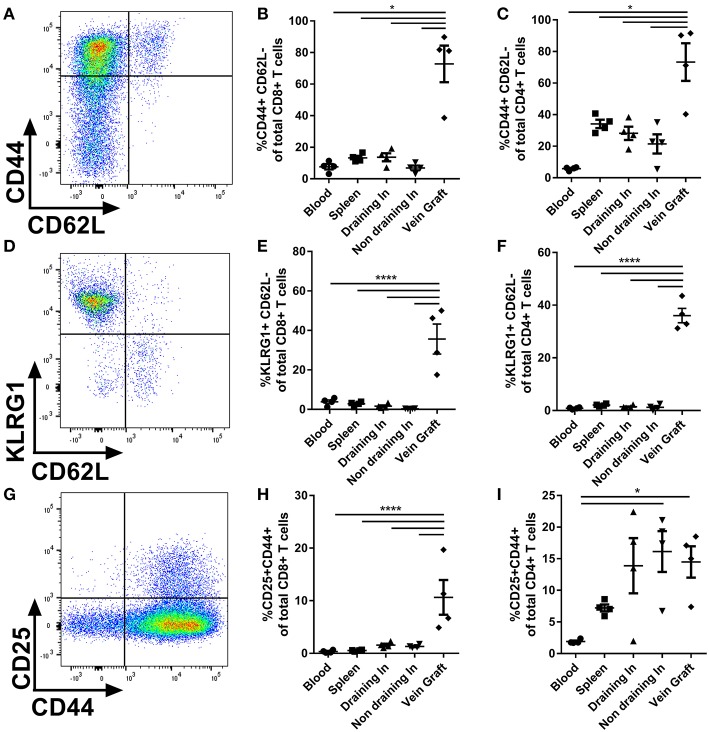
T cell activation in vein grafts. **(A)** Blood, spleen, (non) draining lymph nodes, and vein grafts of C57BL/6 male mice were analyzed with FACS. CD44 and CD62L are used to quantify the percentage of effector T cells. Example of flow cytometry plot of a vein graft sample is shown. **(B)** The percentage of effector CD8+ T cells (CD44+ CD62L- of total CD8+ T cells) is shown. **(C)** The percentage of effector CD4+ T cells (CD44+ CD62L- of total CD4+ T cells) is shown. **(D)** T cell activation was analyzed with KLRG1 and CD62L. Example of flow cytometry plot of a vein graft sample is shown. **(E)** The percentage of activated CD8+ T cells (KLRG1+ CD62L- of total CD8+ T cells) is shown. **(F)** The percentage of CD4+ T cells (KLRG1+ CD62L- of total CD4+ T cells) is shown. **(G)** T cell activation was analyzed with CD25 and CD44. Example of flow cytometry plot of a vein graft sample is shown. **(H)** The percentage of activated CD8+ T cells (CD25+ CD44+ of total CD8+ T cells) is shown. **(I)** The percentage of CD4+ T cells (CD25+ CD44+ of total CD8+ T cells) is shown. **(A–I)** *n* = 4/group. Significant differences between blood, spleen, (non) draining lymph nodes compared to vein grafts are indicated. ^*^P < 0.05, ^****^P < 0.0001, one-way ANOVA and Kruskal-Wallis test.

Next, we examined the activation status of the T cells upon vein graft surgery. CD4+ and CD8+ T cells were phenotypically divided in TN (CD44-CD62L+), effector T cells (CD44+CD62L-), or central memory T cells (TCM) (CD44+CD62L+) (Figure 2A). In vein grafts of control mice we observed a 4-fold increased percentage of CD8+ (Figure 2B) and CD4+ (Figure 2C) effector T cells compared to other organs. Blood, spleen, draining, and non-draining lymph nodes mainly consisted of TN and TCM (Figure S5). The activation status of T cells in vein grafts was validated using the activation markers KLRG1 (23) and CD25. The percentage of KLRG1+CD62L– of total CD8+ and CD4+ T cells in vein grafts, as well as the percentage of CD25+CD44+ of total CD8+ T cells (but not total CD4+ T cells), was strikingly increased compared to blood, spleen, draining, and non-draining lymph nodes (Figures 2D–I). In addition, the percentage of activated effector T cells (CD44+CD62L-KLRG1+ and CD44+CD62L-CD25+) of total CD8+ and CD4+ T cells was significantly higher in vein grafts compared to other organs (Figure S6). A higher percentage of activated T cell in vein grafts compared to the surrounding draining and non-draining lymph nodes indicates local T cell activation in vein grafts. Furthermore, we compared blood, the spleen and non-draining lymph nodes from operated mice with blood, the spleen, and non-draining lymph nodes from naive, not operated mice. No differences were observed between control and naive mice in the percentage of activated T cells (KLRG1+CD62L– of total CD8+ and CD4+ T cells) and effector T cells (CD44+CD62L– of total CD8+ and CD4+ T cells) (Figure S7). This indicated that the vein graft operation on its own had no systemic T cell activation effect. Importantly, the vena cavae that were used as donor grafts showed comparable percentages of CD8+ T cells and CD4+ T cells as vein grafts (Figures S8A,B), but did not contain activated T cells prior to surgery (Figures S8C,D). Together, these data indicate that T cells are locally activated in vein grafts upon surgery without systemic T cell activation.

### CD8+ T Cells Are Instrumental in Preventing Vein Graft Occlusions

**Figure 3:**
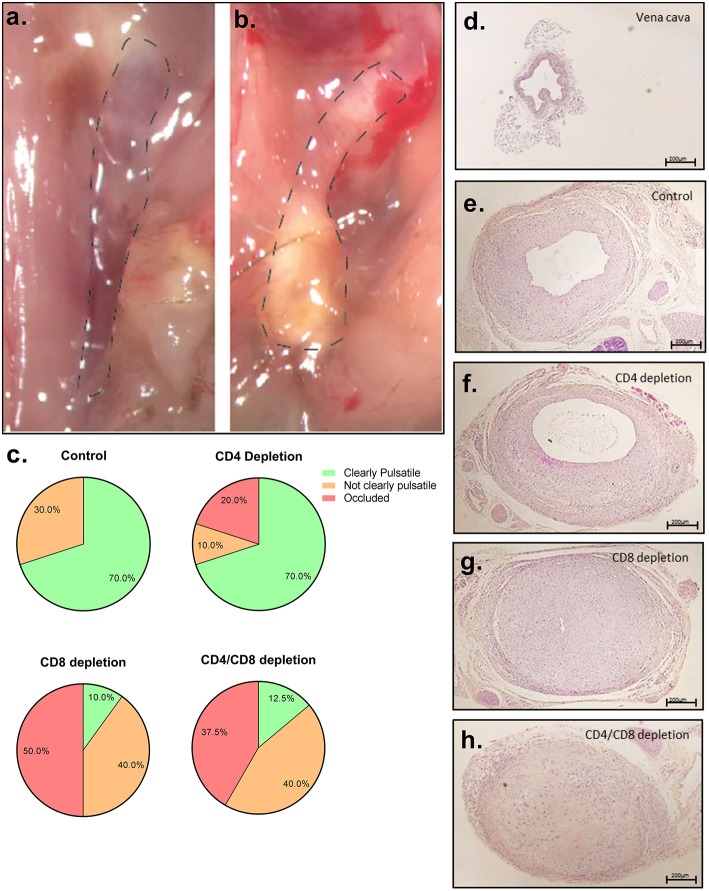
Vein graft patency after T cell depletion. **(A)** Representative picture of a clearly pulsatile vein graft, 28 days after surgery **(B)** and an occluded vein graft. **(C)** Percentage of clearly pulsatile, not clearly pulsatile and occluded vein graft is shown in control (*n* = 10), CD4+ T cell depleted (*n* = 10), CD8+ T cell depleted (*n* = 10), and CD4/CD8+ T cell depleted male mice (*n* = 8). A statistically significant difference with a Kruskal-Wallis test was seen (*p* = 0.04) in the pulsatility of the vein grafts postoperative, with less clearly pulsatile vein grafts in the CD8+ T cell depleted group and in the CD4/8 T cell depleted group compared to the control group (respectively, 70 vs. 10% and 70 vs. 12.5%). Representative pictures are shown of HPS stained **(D)** vena cava prior to operation, **(E)** control vein graft **(F)** vein graft of CD4+ T cell depleted mice **(G)** CD8+ T cell depleted mice and **(H)** CD4/CD8+ T cell depleted mice, 28 days after surgery. 5× magnification, scale bar 200 μm.

**Figure 4:**
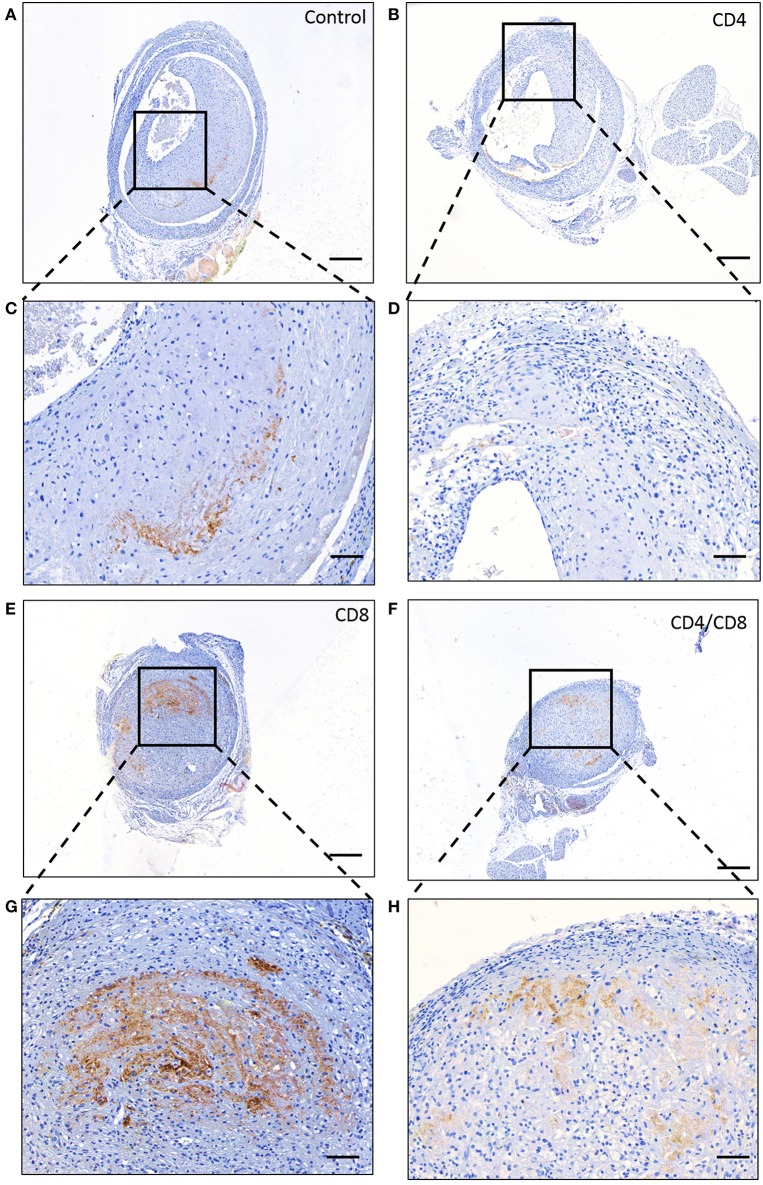
Apoptosis in vein grafts. Representative stainings of caspase 3 in vein grafts of **(A)** control mice, **(B)** CD4+ T cell depleted mice, **(E)** CD8+ T cell depleted male mice and **(F)** CD4/CD8+ T cell depleted mice is shown. 5× magnification, scale bar 200 μm. A zoom in of the vein graft of the concerned **(C)** control mice, **(D)** CD4+ T cell depleted mice, **(G)** CD8+ T cell depleted mice. **(H)** CD4/CD8+ T cell depleted mice is shown. 20× magnification, scale bar 50 μm.

To investigate the particular role of CD4+ and CD8+ T cells in VGD, we depleted these subsets in mice undergoing vein graft surgery, and subsequently investigated vein graft patency. All mice were successfully depleted of either CD4+, CD8+ T cells or both in the blood, spleen, draining, and non-draining lymph node and vein graft after antibody injection at 7 and 14 days after surgery and at sacrifice (Figures S9, S10). Post-operative 28 days after surgery, in both control and CD4+ T cell depleted group 70% of the vein grafts was clearly pulsatile and patent (Figures 3A–F). A statistically significant difference in the pulsatility of the vein grafts post-operative was seen (*p* = 0.04), with less clearly pulsatile vein grafts in the CD8+ T cell depleted group and in the CD4/8 T cell depleted group compared to the control group (respectively, 70 vs. 10% and 70 vs. 12.5%) (Figures 3G,H). Interestingly, in the control group no vein graft were occluded compared to respectively, 50 and 37.5% in the CD8+ T cell depleted group and in the CD4/8 T cell depleted group. Thus, CD8+ T cells mediate a robust protective role in VGD. To provide insight into the mechanism of the CD8+ T cell necessity, we performed a Caspase 3 staining to identify apoptotic cells. In the patent control and CD4 T cell depleted mice, almost no cells in the vein grafts were positive for caspase 3 (Figure 4). In contrast, in the occluded CD8 T cell depleted and CD4/CD8 T cell depleted mice the vein grafts were stained abundantly for caspase 3. So, many cells in the vein grafts lacking CD8+ T cells are apoptotic, suggesting that the absence of CD8+ T cells induces apoptosis in vein grafts, which results in occluded vein grafts.

### TCR Independent T Cell Activation in Vein Grafts

**Figure 5:**
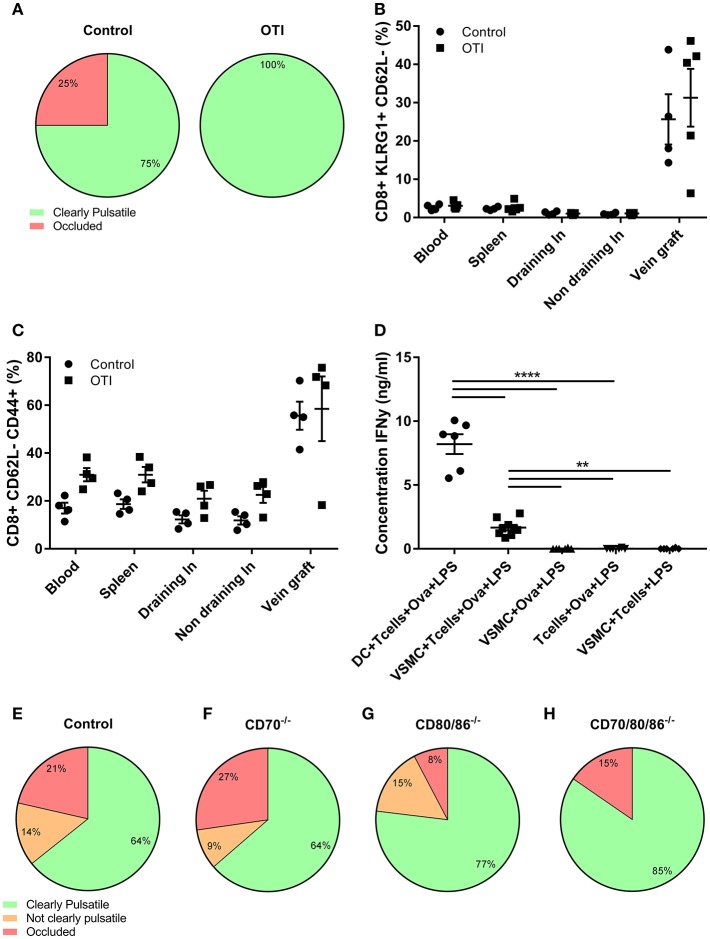
TCR and co-stimulation independent VGD development. **(A)** Vein graft patency of control and OTI male mice is shown. Percentage of clearly pulsatile and occluded vein graft is shown in control and OTI mice, not statistically different (*p* = 0.236). A 2-tailed Student's *t*-test was used. **(B)** The percentage of activated CD8+ T cells (CD8+ KLRG1+ CD62L-), and **(C)** effector CD8+ T cells (CD8+CD62L-CD44+) is shown of control and OTI mice in blood, spleen, (non) draining lymph nodes, and vein grafts. **(A–C)** control *n* = 4, OTI *n* = 5. Mann-Whitney test was used. **(D)** Production of inflammatory cytokines was measured in supernatant of DCs and VMSCs cultured with OTI T cells and Ovalbumin (Ova), stimulated with LPS for 24 h. As control groups, VSMCs were stimulated without OTI T cells or without Ovalbumin and OTI T cell with Ovalbumin were stimulated without VSMCs. The concentration of IFNγ was measured in supernatant. *n* = 6. ^**^P < 0.01, ^****^P < 0.0001. One-way ANOVA was used. **(E)** vein graft patency of control (*n* = 11) and **(F)** CD70^−/−^ (*n* = 12), **(G)** CD80/86^−/−^ (*n* = 14), and **(H)** CD70/80/86^−/−^ (*n* = 14) mice is shown 28 days after surgery. Percentage of clearly pulsatile, not clearly pulsatile and occluded vein graft is shown. A statistically significant difference was not shown (*p* = 0.679), Kruskal-Wallis test was used.

Next, given the local activation of CD8+ T cells in vein grafts and the importance of this T cell subset in the prevention of vein graft occlusions, we aimed to dissect the mechanisms underlying the local stimulation of CD8+ T cells. First, we questioned whether antigenic triggering via the TCR (signal 1) is required for the protective effect of CD8+ T cells in VGD development. For this we used OT-I mice in which all CD8+ T cells bear the same TCR recognizing chicken ovalbumin, an antigen that is not present in mice. Vein graft surgery was performed in these TCR transgenic OT-I mice, and patency of the grafts and T cell activation was determined after 28 days. The vast majority of the control vein grafts (75%) were patent as well as the OT-I mice (100%), which was not statistically different (*p* = 0.236) (Figure 5A). Only one vein graft was occluded in the control group. Moreover, the percentage of KLRG1+CD62L–CD8+ T cells in the control and OT-I mice vein grafts were comparable, and clearly significantly increased compared to other tissues (Figure 5B). In addition, no differences in effector CD44+CD62L–CD8+ T cells between vein grafts of control mice and OT-I mice were observed (Figure 5C). Thus, in presence of a transgenic TCR, CD8+ T cells can still be activated in vein grafts, and CD8+ T cells can still mediate a protective effect in VGD development, indicating that this is TCR independent.

The lack of requirement for TCR triggering may be caused by the absence of cell types able to present antigen to CD8+ T cells. Therefore, we examined if VSMCs, a prominent cell type in vein grafts, can function as APCs to activate T cells via the TCR. VSMCs and DCs, known as professional APCs, were stimulated with LPS in absence or presence of OT-I T cells and Ovalbumin. Albeit less than DCs, VSMCs were capable of T cell activation as evidenced by induction of IFNγ production in OT-I cells (Figure 5D).

### T Cell Co-stimulation Independent VGD Development

T cell co-stimulation cooperates with antigenic triggering. Since we observed that the protective effect of CD8+ T cells in VGD development is independent of TCR triggering, we investigated whether co-stimulation (signal 2) contributes to CD8+ T cell dependent VGD protection. We performed vein graft surgery in mice deficient in one or more co-stimulatory ligands; i.e., CD70^−/−^, CD80/86^−/−^, and CD80/86/70^−/−^ mice. After 28 days, in both control and CD70^−/−^ mice 64% of the vein grafts were clearly pulsatile and patent (Figures 5E,F) and respectively, 77 and 85% of the vein grafts of CD80/86^−/−^ and CD80/86/70^−/−^ mice were clearly pulsatile (Figures 5G,H). A statistically significant difference was not revealed (*p* = 0.679). The pulsatility of the vein grafts, was supported by histology since HPS stained vein grafts showed open grafts and comparable lumen area and total vessel area in all co-stimulation knockout and control mice vein grafts (Figure S11). Only a small decrease in vessel wall area in CD80/86/70^−/−^ mice compared to control mice vein grafts was observed, showing that co-stimulation is not essential for CD8+ T cells to obtain a robust protective effect in vein grafts.

### Cytokines Mediating Bystander CD8+ T Cell Activation Are Present in Vein Grafts

**Figure 6:**
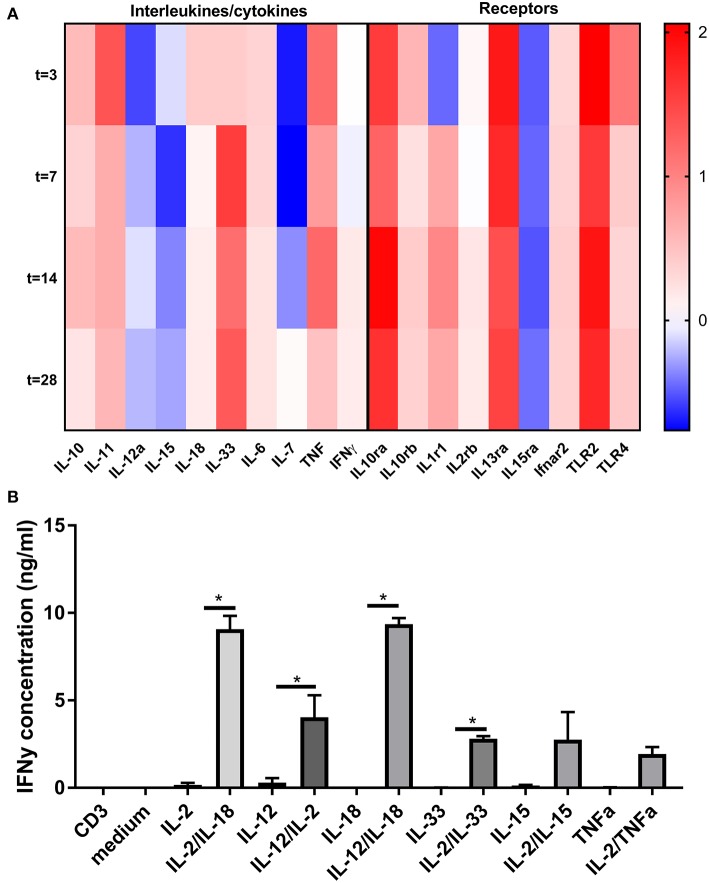
Bystander cytokine presence in vein grafts and contribution to CD8+ T cell activation. **(A)** Heat map of cytokine and cytokine receptor regulation in time in vein grafts. RT^2^ profiler array was performed on RNA isolated of vein grafts in male mice 3, 7, 14, and 28 days after surgery. In total two pools were analyzed (*n* = 3 vein grafts per pool). With the average of these two pools, the 2-ΔΔCt method was used to analyse the relative changes in gene expression. The fold change compared to the control vein graft is shown. **(B)** CD8+ T cells were stimulated for 24 h with agonistic CD3 antibodies, interleukins and TNFα in different combinations. Statistical tests performed compared cytokine combinations to cytokines alone. IFNγ was measured as CD8+ T cell activation marker with ELISA. ^*^P < 0.05, One-way ANOVA was used.

The above described TCR and co-stimulation independent effect of CD8+ T cells in VGD, suggests a role for bystander (non-antigen specific) cytokines (signal 3) of CD8+ T cells in vein grafts. Hence, we examined using the Illumina micro array, whether different cytokines which are capable to provide signals to CD8+ T cells without TCR and co-stimulation activation, are present in vein grafts. Interleukins such IL-6, IL-12a, IL-15, IL18, IL-33, and the effector cytokines TNFα and IFNγ are present and regulated in time in vein grafts after surgery (Figure 6A). Also present are interleukin receptors such as the IL-1, IL-2, and IL-15 receptor. Furthermore, the IFNα and IFNβ receptor Ifnar2 was also up-regulated as well as the Toll-like receptors TLR2 and TLR4, known to induce inflammatory cytokines and up-regulation of co-stimulatory molecules upon ligation.

To investigate CD8+ T cell bystander activation in relation to the plethora of signals present in vein grafts, single, and combinatorial effects of interleukins and co-signals from the TCR were examined *ex vivo* (Figure 6B). Single interleukins such as IL-2, IL-12, IL-15, IL-18, and IL-33 were not capable to elicit IFNγ secretion of CD8+ T cells significantly. Conversely, combinations of interleukins, such as IL-2/IL-18, IL-12/IL-2, IL-12/IL-18, or IL-2/IL-33 induced substantial CD8+ T cell activation as compared to interleukins alone or TCR triggering (mimicked byanti-CD3 monoclonal antibody) alone. Interestingly, CD8+ T cell bystander activation was stronger compared to CD4+ T cell bystander activation (Figure S12). Single interleukins or combinations of interleukins, such as IL-2/IL-18, IL-12/IL-2, or IL-2/IL-33 did not induce IFNγ secretion by CD4+ T cells. Mainly a combination of IL-12 and IL-18 induced CD4+ T cell activation. Collectively, these data indicate that bystander cytokines are present in vein grafts, and can collectively activate CD8+ T cells.

## Discussion

In the current study we show that T cells are modulators of VGD with a specific protective role of CD8+ T cells. Both CD4+ and CD8+ T cells are present in vein grafts and locally activated without systemic activation. Depletion of CD8+ T cells resulted in fully occluded vein grafts, and this was associated with increased apoptosis. Remarkably, the protective effect of CD8+ T cells in vein grafts was TCR and co-stimulation independent. However, bystander signals provided via cytokines can, in a collective manner, activate CD8+ T cells. Importantly, these cytokines are abundantly present in vein grafts.

Previous studies showed that CD8+ T cells modulate cardiovascular diseases (30) and are present in atherosclerotic lesions (31), however, others showed that CD8+ T cells are not functional in atherosclerosis (32, 33). Recent studies have described a role for CD8+ T cells in atherosclerosis, either pro-atherogenic or anti-atherogenic (10). Pro-atherogenic effects of CD8+ T cells (11, 34) have been revealed via their impact on circulating monocyte levels though increased CCL2 and VCAM1 expression, leading to macrophage accumulation in the arterial intima promoting atherosclerotic lesion formation (34). This is in distinction with our results of occlusive vein grafts after depletion of CD8+ T cells. Anti-atherogenic effects of CD8+ T cells have been shown after arterial injury in mice (35). All pro-atherogenic studies described above showed no effect in CD4+ T cell levels after CD8+ T cell depletion, indicating that there is no compensation mechanism of the CD8+ T cell loss by CD4+ T cells. This is in line with our observations in both CD4+ and CD8+ T cell depleted mice where no compensation mechanism after depletion of a T cell subset was observed.

A striking impact of CD8+ T cells was apparent since vein grafts of CD8 T cell depleted mice were occluded. Previous studies showed that CD8+ T cells play a role in anti-tumor immunity (36, 37) and immune checkpoint inhibitors can be effective in the activation of CD8+ T cell responses and can restore anti-tumor immune responses (38). CD8+ T cells in tumors can function via direct and indirect tumor killing. CD8+ T cells are described to recognize target cells via the expression of antigens, which causes direct tumor killing. After recognition, they kill target cells by apoptosis, via the release of perforin and granzymes out of cytotoxic granules. However, CD8+ T cells in vein grafts are activated in an antigen independent manner, which is more similar to indirect tumor killing. Via the release of cytokines, CD8+ T cells can indirectly kill the tumor cells (39, 40). Presence of CD8+ T cells in the tumor environment is associated with beneficial clinical outcomes in several tumor types (37). However, in the studies described above, not only effector CD8+ T cells are described, but also memory CD8 T+ cells that have been previously activated by their cognate antigen are described as indirect tumor killers. Here we observed that many cells in the CD8+ T cell depleted vein grafts and CD4/CD8+ T cells depleted vein grafts are apoptotic, suggesting that the absence of CD8+ T cells induces apoptosis in vein grafts, which results in occluded vein grafts. Thus, CD8 T cells may protect against occlusive lesions by providing survival signals in vein grafts. In addition, CD8^+^ regulatory T cells are also part of the CD8+ T cell subset, although in small subsets, which possess important immunosuppressive functions. However, in this study we did not specifically describe this T cell subset, but CD8^+^ regulatory T cells might be of great value in the protection of VGD by secreting various inhibitory cytokines and chemokines (41).

We questioned whether the protective CD8+ T cell effect was TCR and co-stimulation dependent. In presence of a transgenic TCR, specific for an antigen that is not present in mice, we showed that CD8+ T cells are still activated in vein grafts. Furthermore, vein grafts of OT-I mice were clearly pulsatile, which suggests that the CD8+ T cells can still mediate their protective effect in VGD development, and indicates that the protective CD8+ T cell is TCR independent. *In vivo* we showed no effects of the co-stimulation pathways CD27-CD70 and CD28-CD80/CD86 in VGD development. We showed that vein grafts occlude in CD8+ T cell depleted mice, but in the presence of activated CD8+ T cells, vein grafts are clearly pulsatile. Deficiency of one or both co-stimulatory pathways did not lead to a reduction in vein graft pulsatility, which indicates that the protective effect of CD8+ T cells in VGD development is co-stimulation independent. Interestingly, the vein graft vessel wall area was decreased in *CD70/80/86*^−/−^ mice compared to control mice, suggesting that the protective CD8+ T cell response is stronger than the CD4+ T cell effect. In Figure 6 and Figure S10 we showed that CD8+ T cells can be activated via a plethora of bystander cytokines, in contrast to CD4+ T cells. With the lack of co-stimulation molecules, CD8+ T cells can still be activated via bystander cytokines and may explain the strong CD8+ T cell effect.

We here showed that T cells present in vein grafts are locally activated. Even in the draining lymph nodes, located close to the vein grafts, no activated T cells were detected. The CD8+ T cell activation in vein grafts was found to be TCR independent. In infection models it is clear that TCR triggering is, by definition, essential to activate antigen-specific T cells. T cell activation, however, can also occur in absence of the TCR (42–47). TCR independent CD8+ T cell activation, allows CD8+ T cells to modulate e.g., non-pathogen specific diseases such as VGD, where a vein graft is placed in a sterile environment (18). Here, we describe TCR independent T cell activation in vein grafts of mice, and propose that CD8+ T cell activation in vein grafts is due to bystander signals. Certain cytokines can induce bystander proliferation and activation of CD8+ T cells (18, 48). Especially IL-12 and IL-18 are key regulators of CD8+ T cell activation (49, 50). IFNγ leads to production of IL-12, IL-18, and especially IL-15, which can activate CD8+ T cells (48, 51). CD4+ T cells are mainly activated via a combination of IL12 and IL18. Interestingly, as shown in the heatmap of Figure 6A, IL33 and TNFα are the highest expressed cytokines in vein grafts. However, IL15, IL33, and TNFα could not activate CD4+ T cells (Figure S12), in contrast to CD8+ T cells (Figure 6B). Type I interferons, IFNα, and IFNβ also showed to enhance IFNγ production, synergistically with IL-18 in human T cells (52). In contradiction, CD8+ T cell inhibition via type I Interferons was shown via suppression of the IL-12 pathway (53). Several other cytokines, including IL-1β and TNF-α, can enhance IFN-γ production in CD8+ T cells (18, 54). IL-33 is mainly expressed by Th2 cells and mast cell, but IL-33 has also been shown to enhance TCR independent virus specific CD8+ T cell activation (18, 55). However, in line with our results, these cytokines do not activate CD8+ T cells individually, but combinations of cytokines are functional and show synergic effects.

However, it should be taken in to account that VGD is a complex progress involving multiple components, besides CD8+ T cells. As indicated, non-T cells including macrophages, DCs and B cells represent around 75% of the graft infiltrate. It was previously shown that these cells contribute to the vein graft outcome. In the current study we showed a protective effect of CD8+ T cells in VGD via bystander cytokines. However, this effect can be either directly via bystander cytokines or indirectly via the activation of innate immune cells via bystander cytokines. In this study, we mainly focused on the direct effect of CD8+ T cells. However, in the pathophysiology of VGD, both innate and immune cells are involved.

In conclusion, we here show that T cells play a role in VGD, with a specific protective role of CD8+ T cells. The protective effect of CD8+ T cells in vein grafts is TCR and co-stimulation independent. Bystander cytokines are abundantly present in vein grafts and have the capacity to activate CD8+ T cells in a collective manner independent of TCR and co-stimulation signals. This gives rise to potential new strategies to use T cell bystander cytokines in a therapeutic approach to prevent VGD.

## Data Availability

All datasets generated for this study are included in the manuscript and/or the Supplementary Files.

## Ethics Statement

This study was performed in compliance with Dutch government guidelines and the Directive 2010/63/EU of the European Parliament. All animal experiments were approved by the animal welfare committee of the Leiden University Medical Center.

## Author Contributions

KS, HP, MV, JJ, PQ, and RA: designing research studies. KS, HP, and RA: conducting experiments. KS and HP: acquiring data. KS, RA, MV, JJ, and PQ: analyzing data. RA, JJ, and PQ: providing reagents. KS, RA, PQ, and MV: writing the manuscript.

### Conflict of Interest Statement

The authors declare that the research was conducted in the absence of any commercial or financial relationships that could be construed as a potential conflict of interest.
