# Supplementary material for: CD8+ T Cells Protect During Vein Graft Disease Development
Source: Front Cardiovasc Med. 2019 Jun 13;6:77. doi: 10.3389/fcvm.2019.00077 (PMC6584838; doi:10.3389/fcvm.2019.00077)
Supplement: Supplementary file 1 [file Data_Sheet_1.pdf]

Supplemental figures

Figure S1. TCR transgenic OT-I mouse

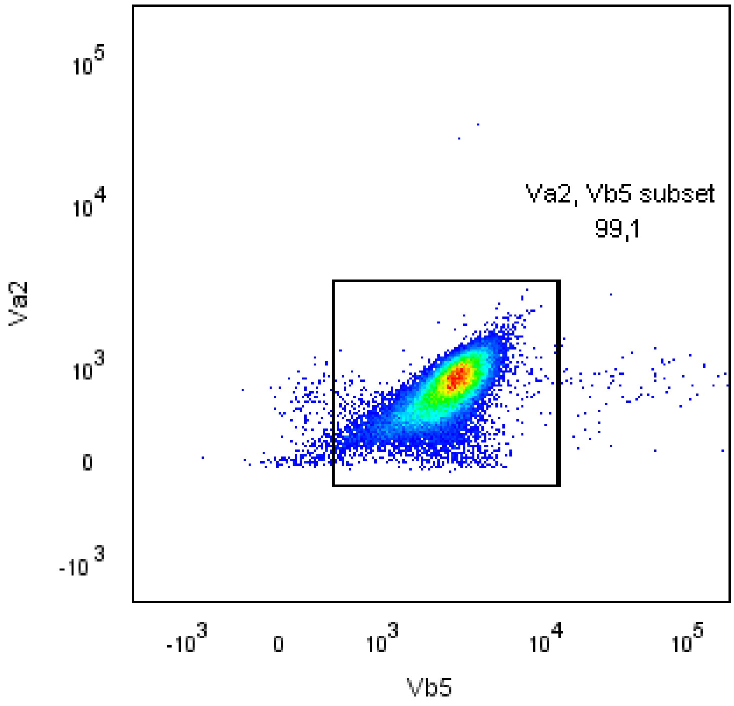

Figure S1. Expression of Va2 and Vb5 on splenic CD3+CD8+ cells of OT-I male mice. Percentage is shown.

Figure S2. Flow cytometry gating strategies.

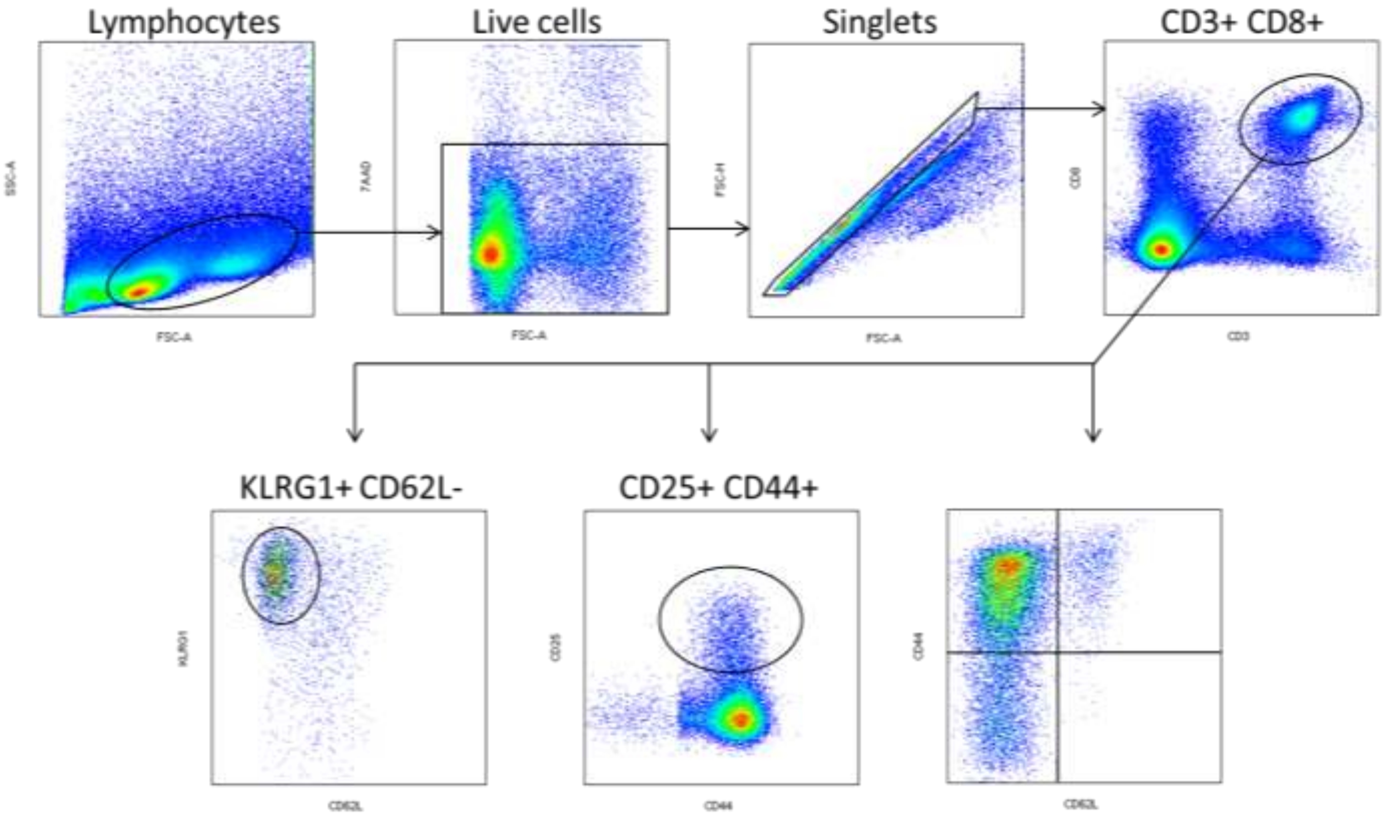

Figure S2. Flow cytometry gating strategies. Representative plots show the gating strategy for detecting activated T cells and effector, central and naive T cells as depicted in figure 1,2 and 4. In this sequential gating, cells were first gated on lymphocytes (forward-scatter (FSC-A) vs. side-scatter (SSC-A)). The cells were analysed for their uptake of 7AAD to exclude dead cells and were subsequently gated on singlets (FSC-A vs. FSC-H). The cells were further analysed on their CD3 and CD8 expression. CD3+ CD8+ T cells were gated on KLRG1, CD62L, CD44 and CD25.

Figure S3. Infiltrating CD3+ T cells in vein grafts.

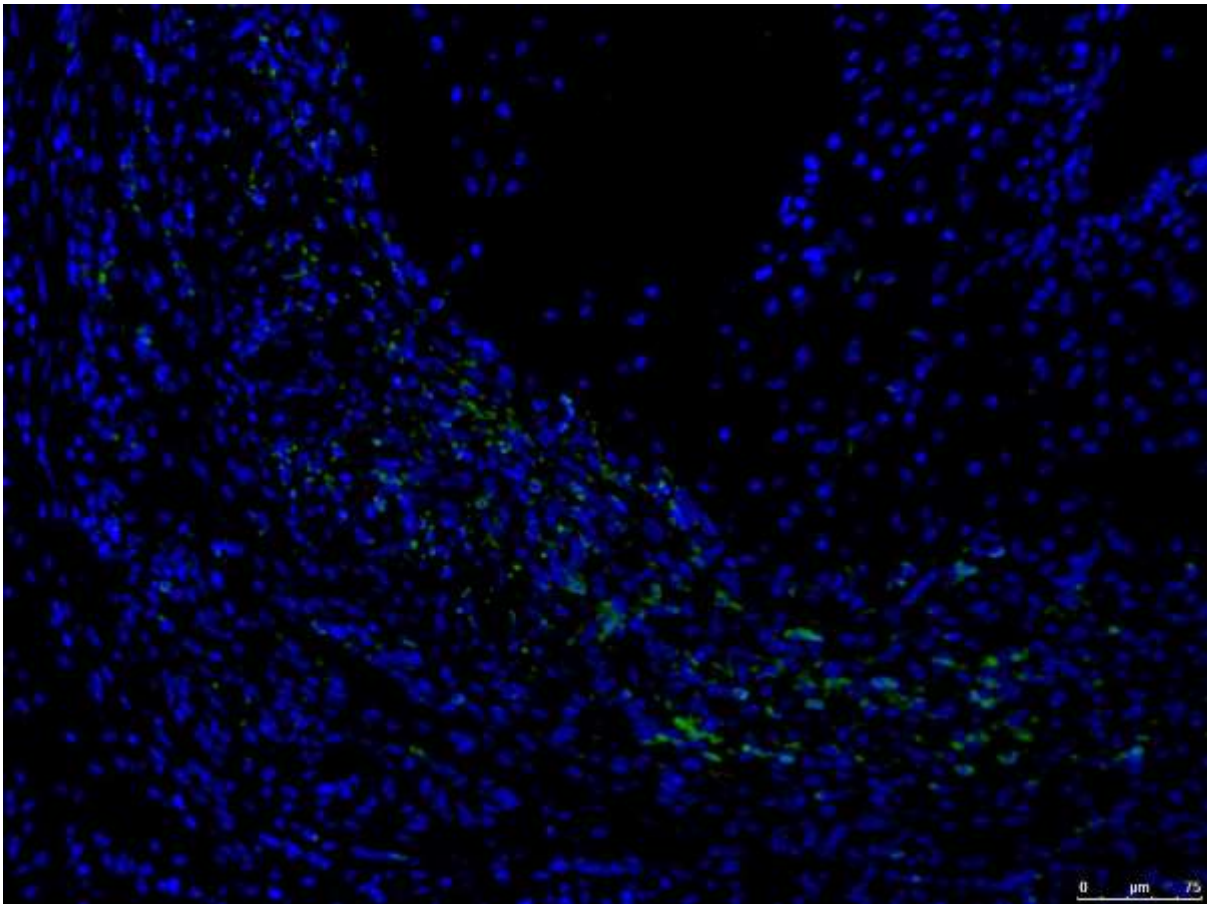

Figure S3. Representative immunofluorescent staining showing CD3+ T cells (green) in a vein graft of a control C57BL/6 mice. DAPI (blue) is used to stain the DNA of cells. Scale bar 75µm.

Figure S4. Composition of blood, spleen, draining and non-draining lymph nodes.

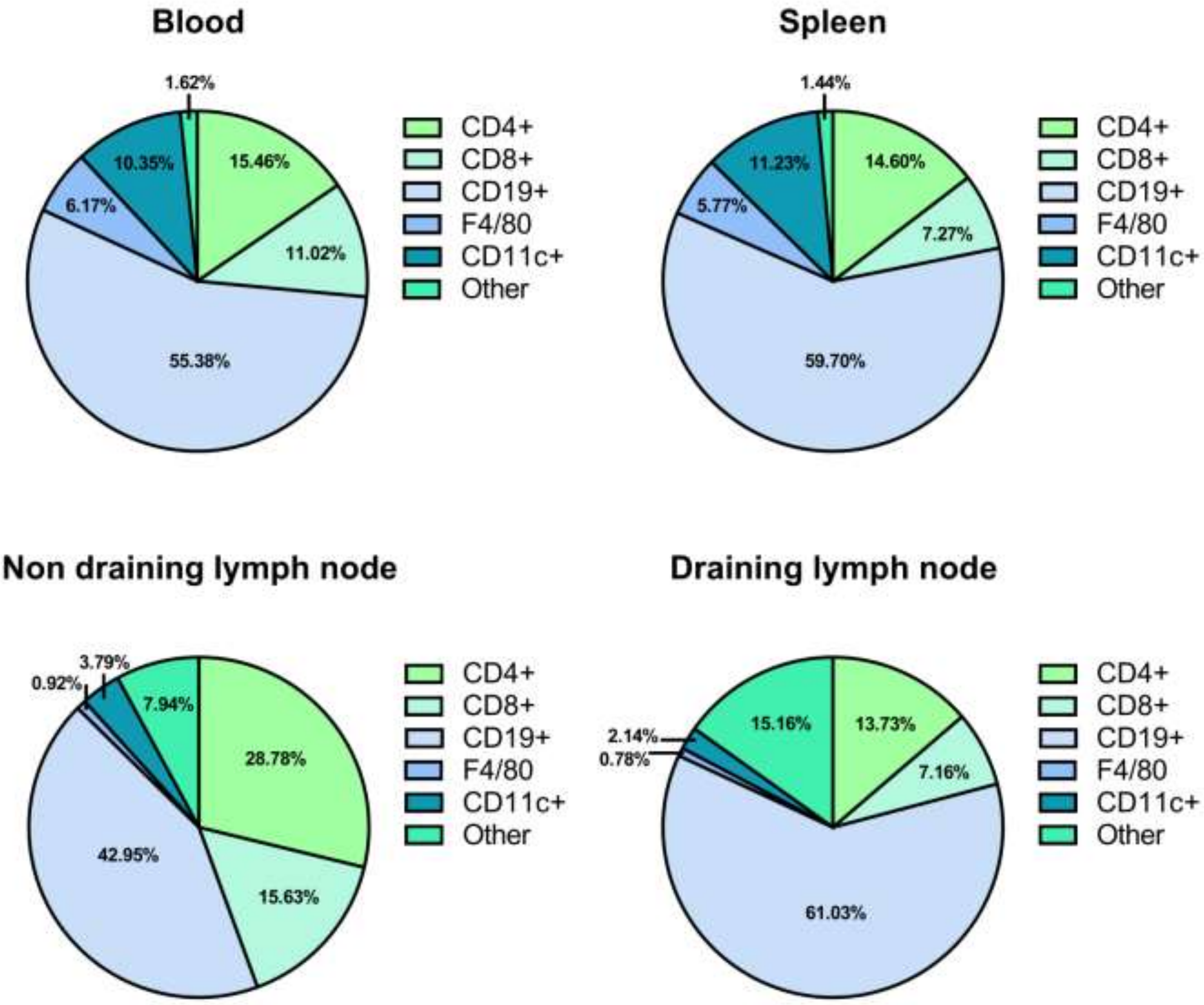

Figure S4. Cell composition measured with FACS analysis. Percentage of CD4+, CD8+, CD19+, F4/80+, CD11c+ and other cells are shown in **a.** blood **b.** spleen **c.** non-draining lymph node and **d.** draining lymph node. Percentage is shown. n=4/group.

Figure S5. Central memory and naive T cells in vein grafts.

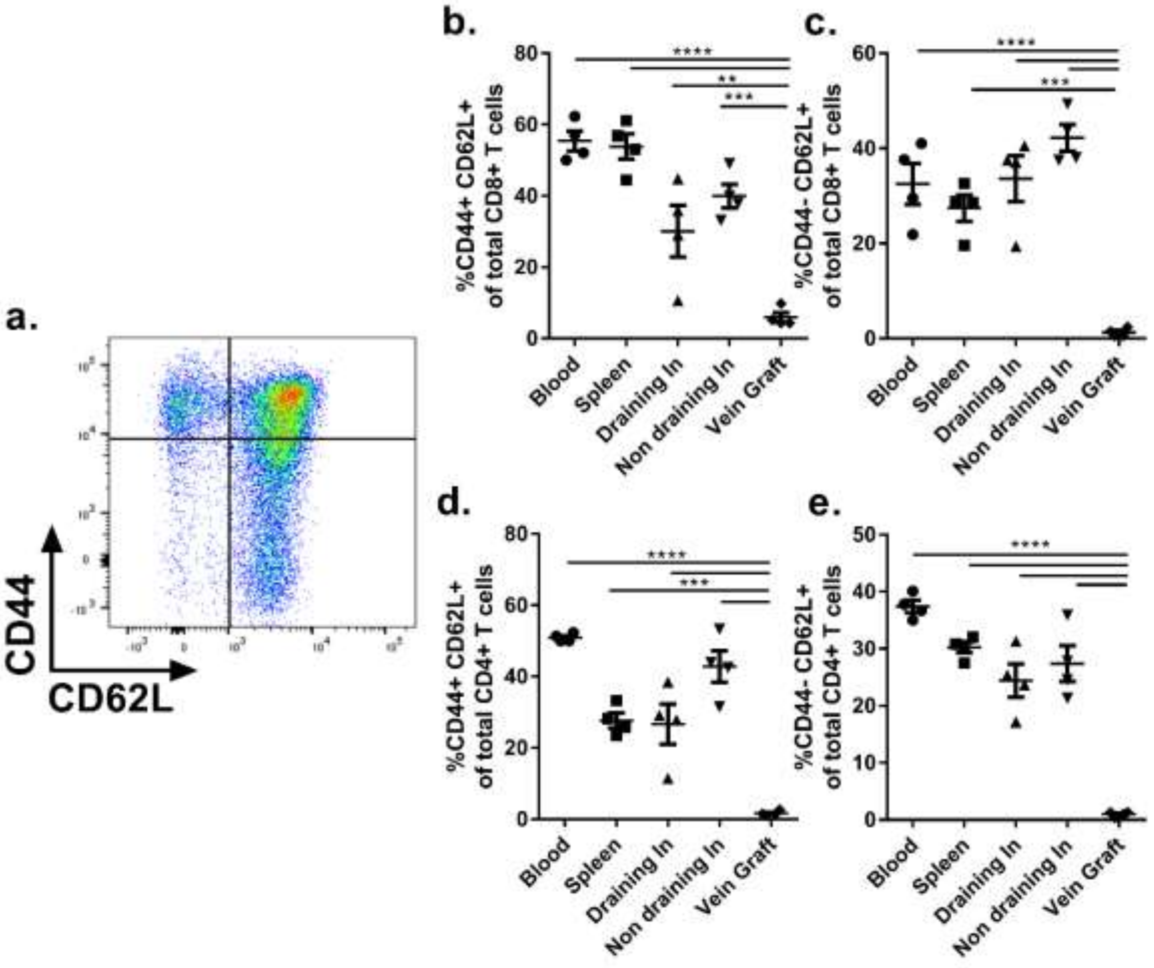

Figure S5a. Blood, spleen, non-draining and draining lymph nodes and vein grafts of male mice were analysed with FACS. CD44 and CD62L are used to quantify the percentage of central memory T cells and naïve T cells. Example of flow cytometry plot is shown of a blood sample. **b.** The percentage of central memory CD8+ T cells (CD44+ CD62L+ of total CD8+ T cells) is shown. **c.** The percentage of naive CD8+ T cells (CD44- CD62L+ of total CD8+ T cells) is shown. **d.** The percentage of central memory CD4+ T cells (CD44+ CD62L+ of total CD4+ T cells) is shown. **e.** The percentage of naive CD4+ T cells (CD44- CD62L+ of total CD8+ T cells) is shown. b-e n=4/group. Significant differences between blood, spleen, (non) draining lymph

nodes compared to vein grafts are indicated. *p<0.05, **p<0.01, ***p<0.001, ****p<0.0001. One-way ANOVA test was used.

Figure S6. Effector T cell activation

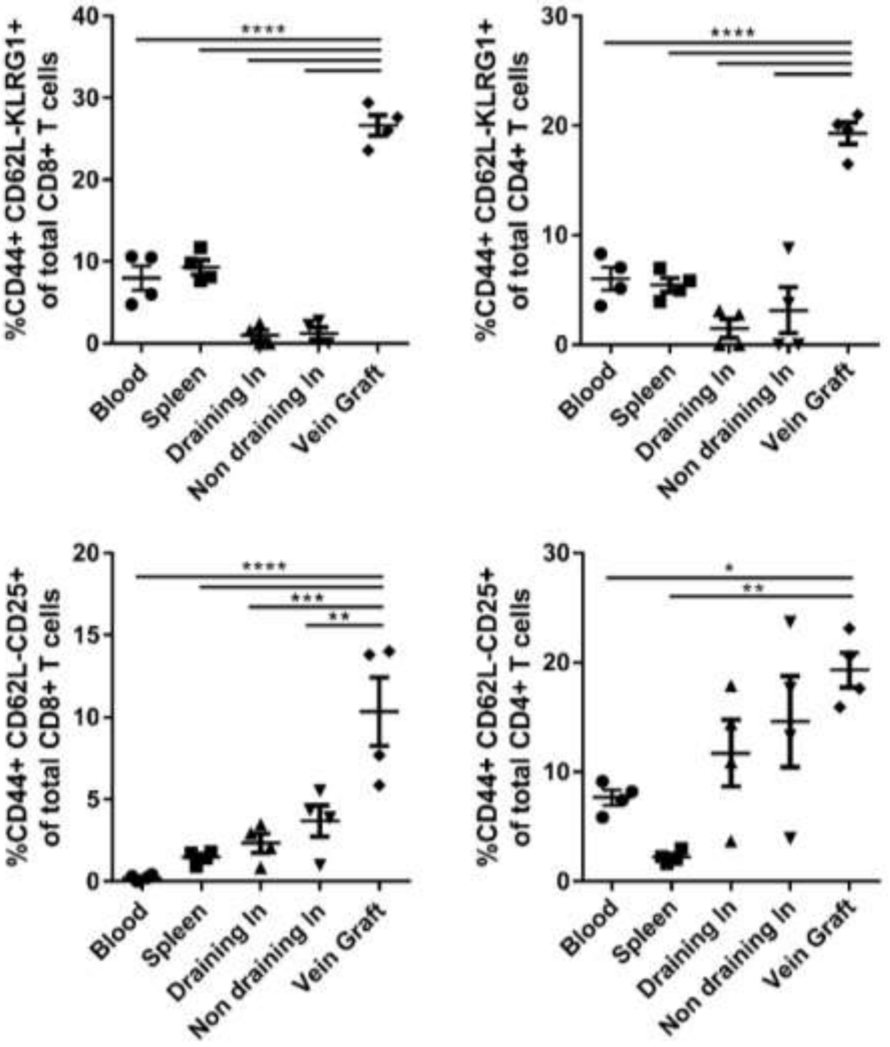

Figure S6. Blood, spleen, (non) draining lymph nodes and vein grafts of male mice were analysed with FACS. CD44 and CD62L are used to quantify the percentage of effector T cells. T cell activation was analysed with KLRG1 and CD25. **a.** The percentage of activated effector CD8+ T cells is shown as CD44+ CD62L-KLRG1+ of total CD8+ T cells. **b.** The percentage of activated effector CD4+ T cells is shown as CD44+ CD62L-KLRG1+ of total CD4+ T cells. **c.** The percentage of activated effector CD8+ T cells is shown as CD44+ CD62L-CD25+ of total CD8+ T cells. **d.** The percentage of activated effector CD4+ T cells is shown as CD44+ CD62L-CD25+ of total CD4+ T cells. 1-d n=4/group. Significant differences between blood,

spleen, (non) draining lymph nodes compared to vein grafts are indicated. *p<0.05, **p<0.01, ***p<0.001, ****p<0.0001. One-way ANOVA test was used.

Figure S7. T cell activation in blood, the spleen and non-draining lymph nodes of control and naive mice.

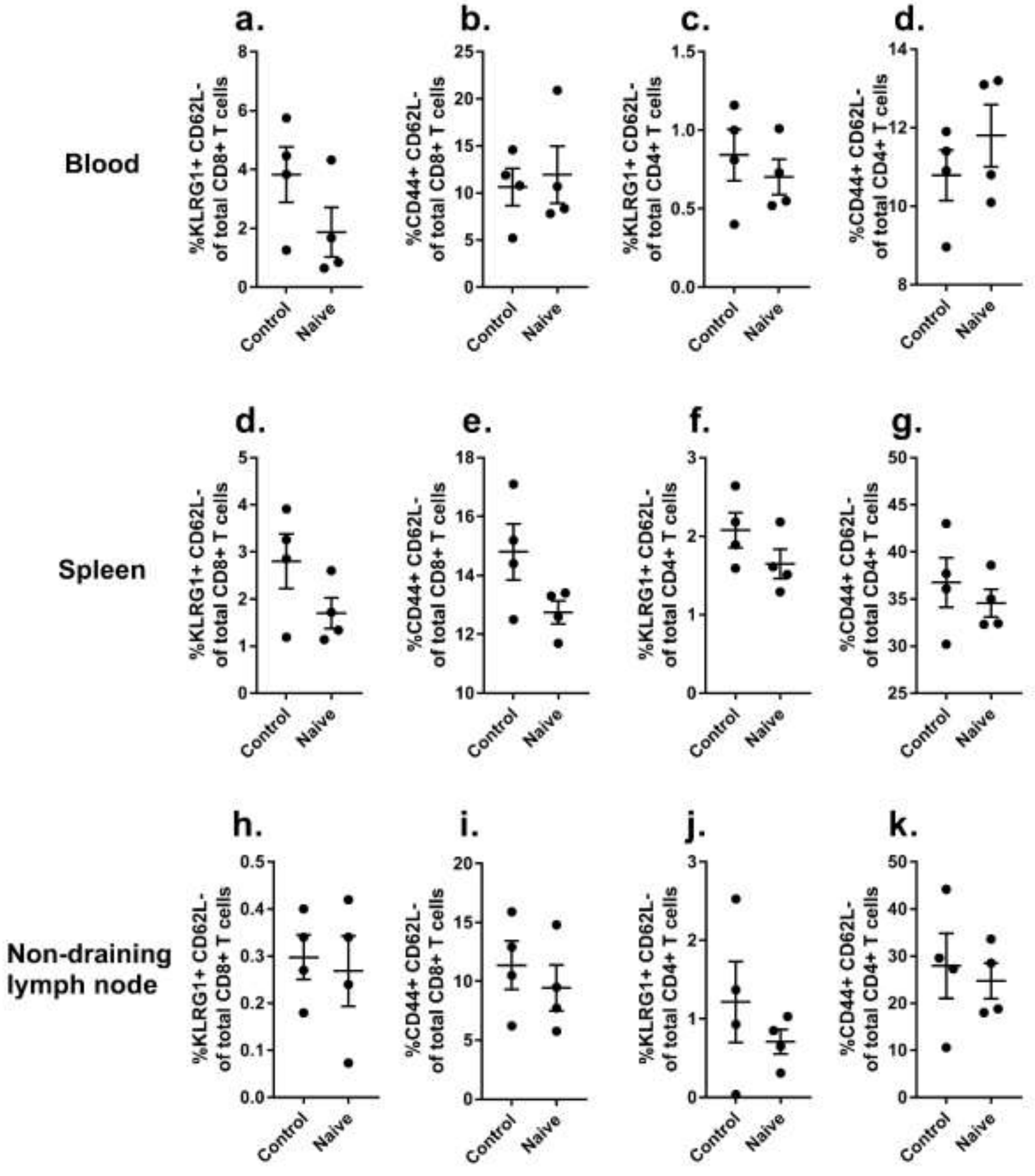

Figure S7. Blood, the spleen and non-draining lymph nodes of control male mice and naive male mice were analysed with FACS. KLRG1 and CD62L are used to quantify the percentage activated T cells. **a.** percentage of KLRG1+CD62L- of total CD8+ T cells **b.** percentage of CD44+CD62L- of total CD8+ T cells **c.** percentage of

KLRG1+CD62L- of total CD4+ T cells **d.** percentage of CD44+CD62L- of total CD4+ T cells. Percentage is shown. a-k n=4/group. Mann-Whitney test was used.

Figure S8. T cell percentage and activation in vena cava and vein graft.

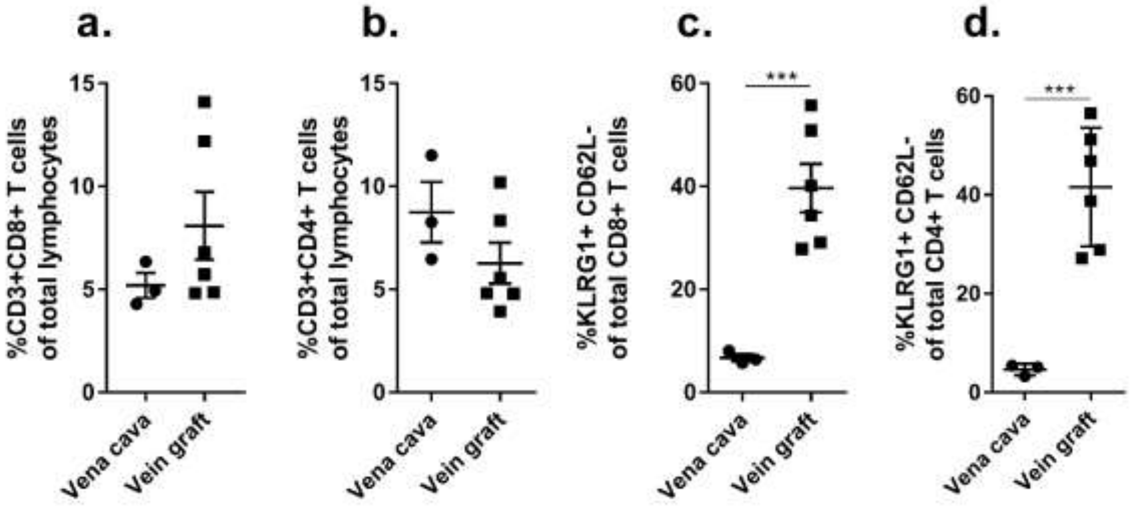

Figure S8a. Vena cava and vein grafts of C57Bl/6 male mice were analysed with FACS. Percentage of CD3+CD8+ T cells of total lymphocytes is shown and **b.** percentage of CD3+CD4+ T cells of total lymphocytes. **c.** percentage of KLRG1+CD62L- of total CD8+ T cells **d.** percentage of KLRG1+CD62L- of total CD4+ T cells. Percentage is shown. a-d n=4/group. *p<0.05, **p<0.01, ***p<0.001. Mann-Whitney test was used.

Figure S9. T cell depletion in blood.

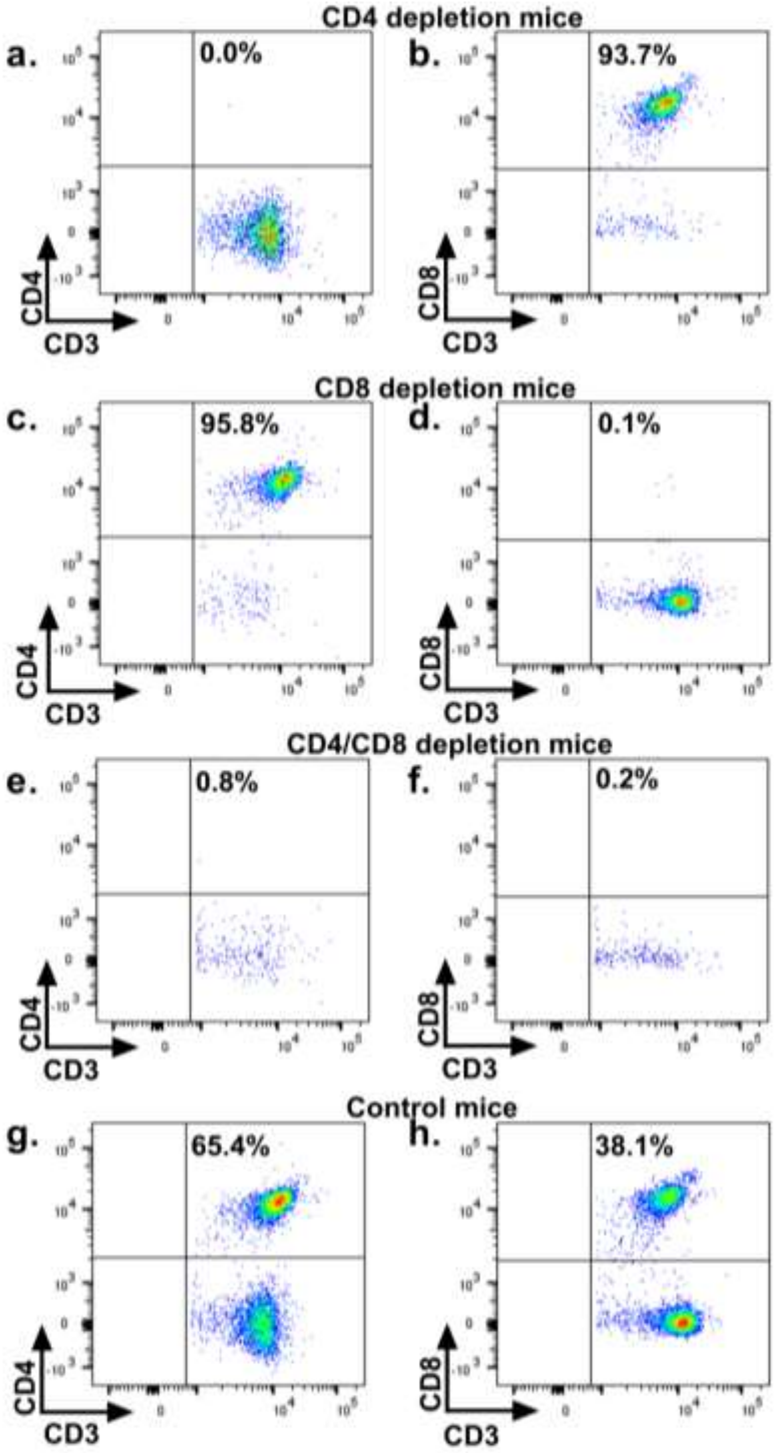

Figure S9. T cell depletion in blood. Example of flow cytometry plot is shown of **a.** CD4+ T cells and **b.** CD8+ T cells in CD4 T cell depleted mice. **c.** CD4+ T cells and **d.** CD8+ T cells in CD8+ T cell depleted mice. **e.** CD4+ T cells and **f.** CD8+ T cells in

CD4/CD8+ T cell depleted male mice. **g.** CD4+ T cells and **h.** CD8+ T cells in control mice.

Figure S10. T cell depletion in blood, spleen, draining lymph node, non-draining lymph node and vein graft.

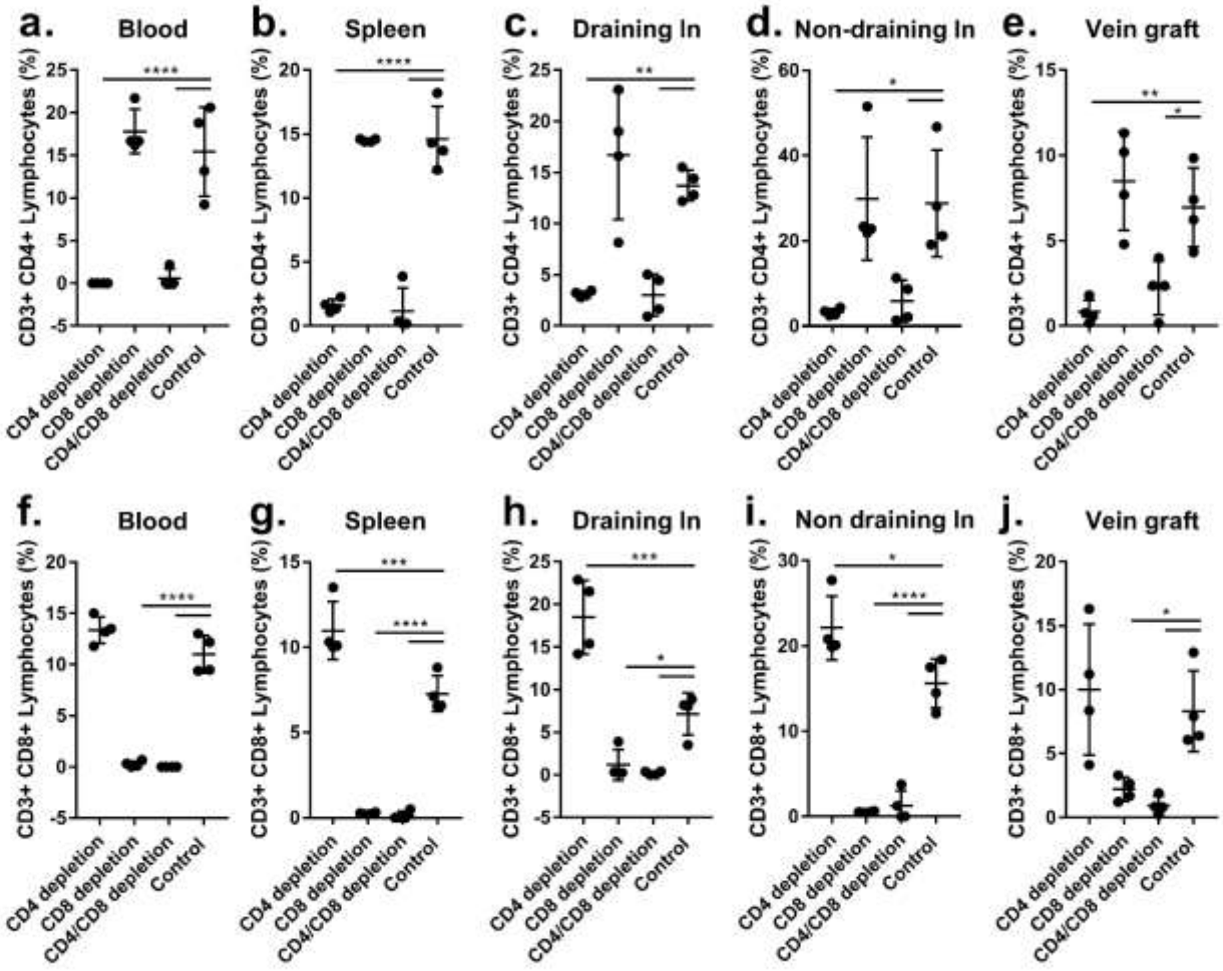

Figure S10. T cell depletion in blood, spleen, draining lymph node (ln), non-draining ln and vein graft of male mice. Percentage is shown of CD3+ CD4+ T cells in CD4 T cell depleted mice, CD8+ T cell depleted mice, CD4/CD8+ T cell depleted mice and control mice in **a.** blood, **b.** spleen, **c.** draining lymph node **d.** non-draining lymph node **e.** vein graft. Percentage is shown of CD3+ CD8+ T cells in CD4 T cell depleted mice, CD8+ T cell depleted mice, CD4/CD8+ T cell depleted mice and control mice in **f.** blood, **g.** spleen, **h.** draining lymph node **i.** non-draining lymph node

j. vein graft. n=4/group. *p<0.05, **p<0.01, ***p<0.001, ****p<0.0001. One-way ANOVA test was used.

Figure S11. Vein graft composition of co-stimulation knockout mice.

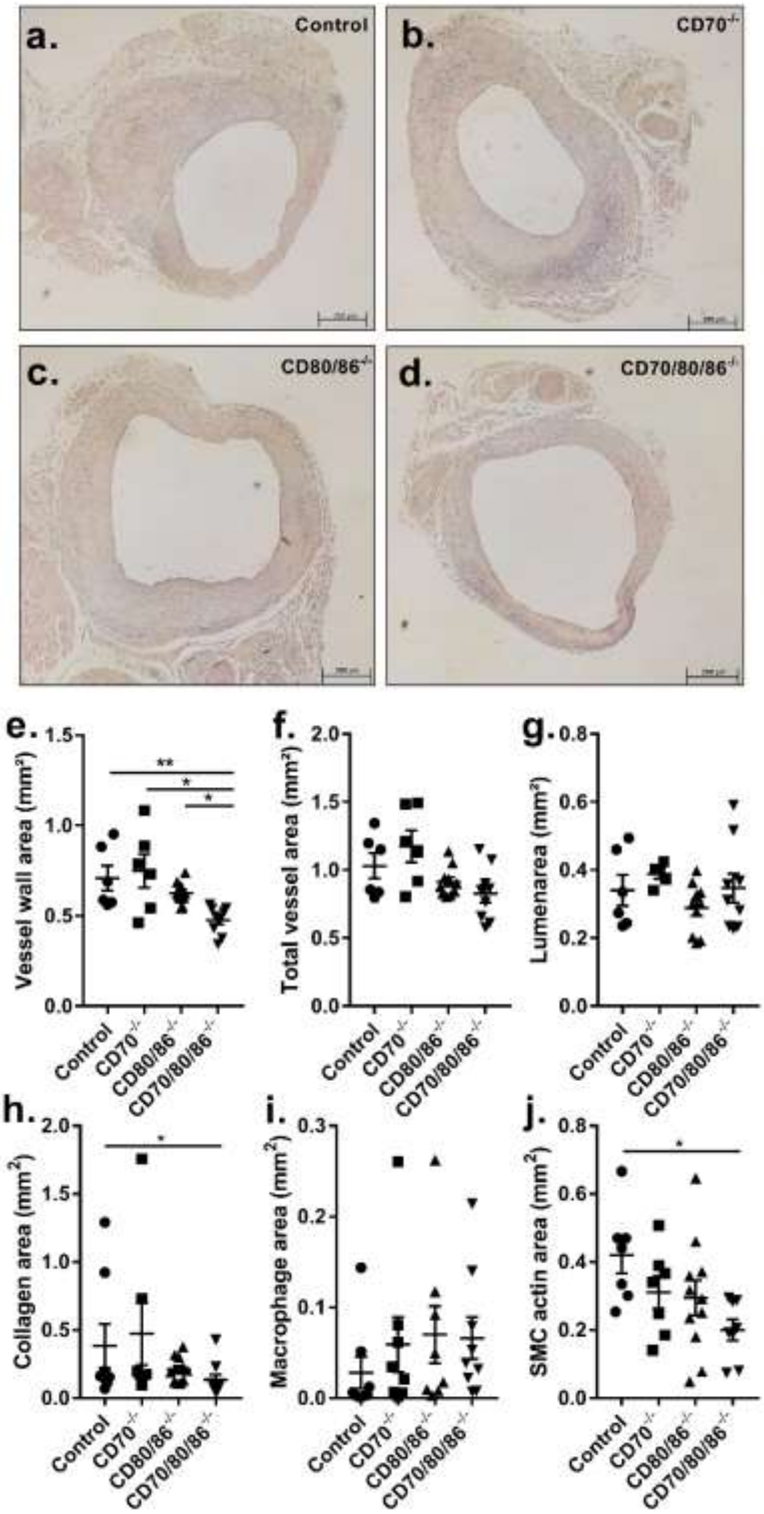

Figure S11. Vein graft composition of co-stimulation knockout male mice. **a.** Representative pictures of HPS stained vein graft 28 days after surgery is shown of control mice, **b.** CD70$^{-/-}$ mice, **c.** CD80/86$^{-/-}$ mice, **d.** CD70/80/86$^{-/-}$ mice. 5x

magnification, scale bar 200 µm. **e.** Vessel wall area (mm^2) in vein grafts 28 days after surgery, **f.** Total vessel area (mm^2) and **g.** lumen area (mm^2) in control, $CD70^{-/-}$, $CD80/86^{-/-}$ and $CD70/80/86^{-/-}$ mice is shown. (Immuno)histochemical staining of vein grafts is shown of **h.** Collagen area (mm^2), **i.** Macrophage area (mm^2), **j.** SMC area (mm^2). Control (n=10), $CD70^{-/-}$ (n=8), $CD80/86^{-/-}$ (n=11) and $CD80/86/70^{-/-}$ (n=12) mice. *p < 0.05, **p < 0.01, Kruskal-Wallis test was used.

Figure S12. Bystander cytokine contribution to CD4+ T cell activation.

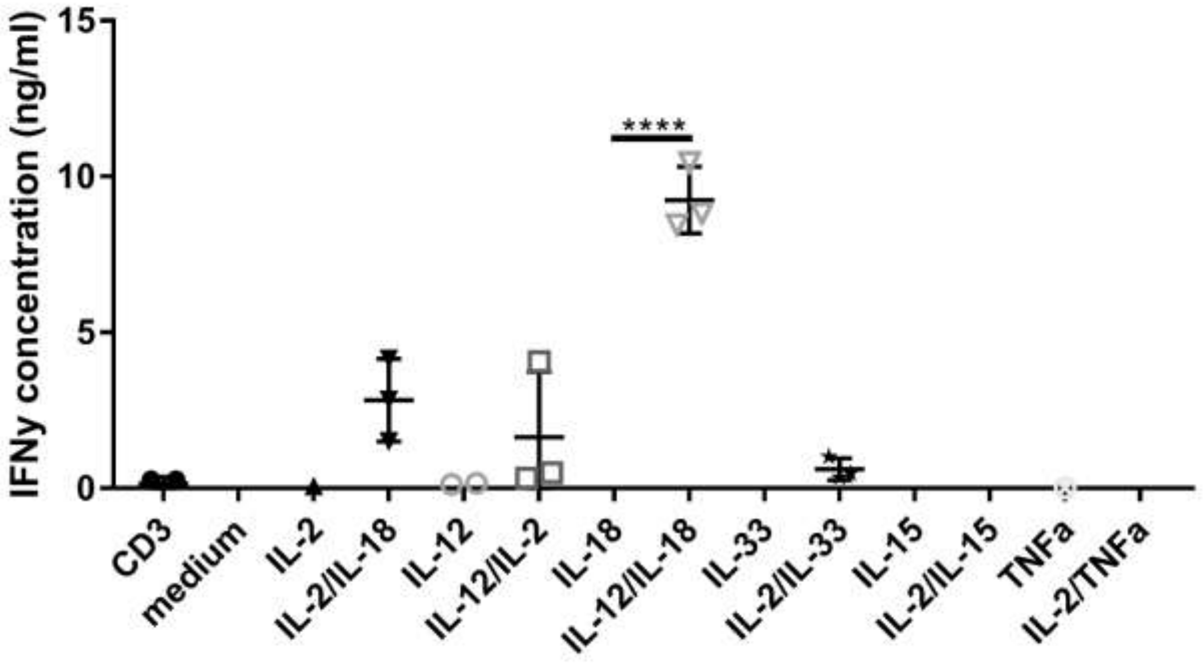

Figure S12. Bystander cytokine contribution to CD4+ T cell activation. CD4+ T cells were stimulated for 24 hours with agonistic CD3 antibodies, interleukins and TNFα in different combinations. Statistical tests performed compared cytokine combinations to cytokines alone. IFNγ was measured as CD4+ T cell activation marker with ELISA. n=3/group. ****$p<0.0001$, One-way ANOVA was used.
